# Assessment of change in drug-induced parkinsonism in daily-life gait using vision-based machine learning

**DOI:** 10.1371/journal.pdig.0001780

**Published:** 2026-10-15

**Authors:** Benjamin Filtjens, Vida Adeli, Soroush Mehraban, Andrea Sabo, Babak Taati, Andrea Iaboni

**Affiliations:** 1 Department of Engineering Systems and Services, Delft University of Technology, Delft, the Netherlands; 2 Institute for Health Systems Science, Delft University of Technology, Delft, the Netherlands; 3 KITE Research Institute, University Health Network, Toronto, Ontario, Canada; 4 Vector Institute, Toronto, Ontario, Canada; 5 Department of Computer Science, University of Toronto, Toronto, Ontario, Canada; 6 Institute of Biomedical Engineering, University of Toronto, Toronto, Ontario, Canada; 7 Department of Psychiatry, University of Toronto, Toronto, Ontario, Canada; The University of British Columbia, CANADA

## Abstract

Drug-induced parkinsonism is a potentially reversible movement disorder that may be subtle and difficult to monitor in residential dementia care. Vision-based machine learning analysis of gait may provide unobtrusive longitudinal monitoring around medication changes. We conducted a two-center cohort study of 61 residents with dementia receiving psychotropic medication. Hallway gait sequences were recorded using ceiling-mounted cameras and converted to three-dimensional body pose. A TabPFN classifier estimated UPDRS-III item 29 gait severity for each walk. We evaluated associations between acute antipsychotic (AP) and antidepressant (AD) dose reduction/cessation (≥50% dose reduction or cessation, 0–21 days after change) and daily mean predicted gait severity using linear mixed-effects models in ever-exposed participants, with complementary equal-weight paired analyses among participants observed in both medication states. In mixed-effects models, acute AP dose reduction/cessation was associated with lower predicted gait severity (β=−0.127, *p* = 0.022), whereas AD dose reduction/cessation was not (β=−0.060, *p* = 0.210). Equal-weight paired analyses found no statistically significant participant-average within-person change among paired participants (AP mean change = 0.031, 95% CI [−0.114, 0.176], *p* = 0.653; AD mean change =−0.021, 95% CI [−0.098, 0.056], *p* = 0.575), consistent with heterogeneous individual responses. Vision-based monitoring captured within-participant gait variation around psychotropic dose-reduction periods, but findings are associative. Larger prospective studies with documented reasons for medication changes are needed before using such monitoring to guide medication decisions.

## Author summary

Drug-induced parkinsonism is a movement disorder that can occur when older adults take medications used for psychiatric or behavioral symptoms. It can be difficult to detect because symptoms may be subtle and develop gradually, while clinical assessments are often infrequent. We studied whether a camera-based monitoring system installed in residential-care hallways could help track walking changes after medication dose reductions. The system turns short walking videos into body-pose measurements and uses these measurements to estimate gait severity. In 61 residents with dementia, we found that antipsychotic dose reduction was linked with lower estimated parkinsonism gait severity in a participant-day mixed-effects analysis. However, when we gave each paired participant one equal-weight contrast among participants observed both on medication and after a dose reduction/cessation event, we did not find a clear average improvement, and individual responses varied substantially. Antidepressant dose reduction showed no consistent pattern. Our findings suggest that unobtrusive gait monitoring may help identify people whose walking changes around medication adjustments, but it should not be used alone to guide medication decisions.

## Introduction

Drug-induced parkinsonism (DIP) is a common adverse effect of psychotropic medications and frequently goes undetected or is recognized late because early symptoms can be subtle, the onset is gradual, and routine clinical assessments are episodic [1,2]. In older adults, parkinsonism increases the risk of falls and mobility decline, and symptoms may improve after therapeutic adjustments such as dose reduction or switching agents [3]. These clinical features make DIP an important medication-safety problem in residential dementia care, where psychotropic medications are commonly used [4,5]. Assessment can be particularly challenging in this population, as performance-based mobility assessments may be difficult to administer in people with dementia [6].

Current approaches to gait assessment include structured clinical observation, wearable sensors, and instrumented testing [7]. Although informative, structured assessments provide only brief snapshots of gait, while instrumented approaches may require dedicated equipment or the use of wearable devices [7]. Ambient computer-vision systems offer a complementary strategy by measuring walking during routine movement through the care environment. Previous work has shown that unobtrusive hallway video can quantify mobility, fall risk, and parkinsonian gait in older adults with dementia [8–10]. The remaining evidence gap is whether such daily-life gait estimates are useful for medication-linked longitudinal monitoring, where the objective is to detect within-person changes around real-world medication adjustments.

This study evaluated a vision-based gait-severity pipeline for longitudinal monitoring around psychotropic dose reduction or cessation in two residential dementia-care cohorts. We linked repeated daily-life gait estimates with medication administration records to examine associations between antipsychotic and antidepressant dose reduction/cessation and predicted parkinsonian gait severity. We hypothesized that psychotropic dose reduction or cessation would be associated with lower predicted gait severity, while recognizing that observational medication changes are confounded by clinical indication and may produce heterogeneous individual trajectories.

## Materials and methods

### Ethics statement

Ethical approval was granted by the University Health Network Research Ethics Board (CAPCR IDs 24–5835, 15–9693, and 18–5459). Written informed consent was obtained from substitute decision-makers, and assent was obtained from all participants. The study was conducted in accordance with applicable institutional human-participant research requirements and the principles of the Declaration of Helsinki.

### Data acquisition

Data were collected from participants admitted to a Specialized Dementia Unit (T-SDU; a psychogeriatric unit for people with dementia and behavioral symptoms) and a Long-Term Care facility (T-LTC) in Toronto, Canada. Inclusion criteria were a confirmed dementia diagnosis (based on geriatric psychiatric assessment) and the ability to independently walk at least 20 meters. Exclusion criteria were a diagnosis of idiopathic Parkinson’s disease and requiring a walking aid.

Clinical measurements were taken at baseline and included demographic information (age and sex), severity of neuropsychiatric and cognitive symptoms assessed using the Neuropsychiatric Inventory (NPI) total score [11] and the Severe Impairment Battery-Short Form (SIB-S) [12], and independence in daily activities assessed by the Katz Index [13]. Administration and dosing of antipsychotics (AP) and antidepressants (AD) were extracted from medical records. Gait sequences recorded on days without medication administration records were excluded from analyses.

### Parkinsonian gait severity monitoring

Gait was monitored using a ceiling-mounted RGB camera (1920 × 1080 px) recording video at 30 frames per second (Fig 1). Participants wore RFID tags that automatically triggered recording when walking through the monitored hallway [8–10]. In routine deployment, the video stream is segmented into candidate walking bouts using the hallway camera field of view and RFID/trajectory information, after which non-walking behavior can be filtered before gait scoring. For this retrospective analysis, all recordings were manually reviewed and only uninterrupted straight-walking segments were retained, excluding turns, intentional stops, social interactions, and other non-walking behaviors. The RGB video stream was processed using WHAM [14], a validated monocular mesh recovery algorithm, to estimate three-dimensional body pose based on the SMPL body model [15] (24 joints per frame).

**Fig 1:**
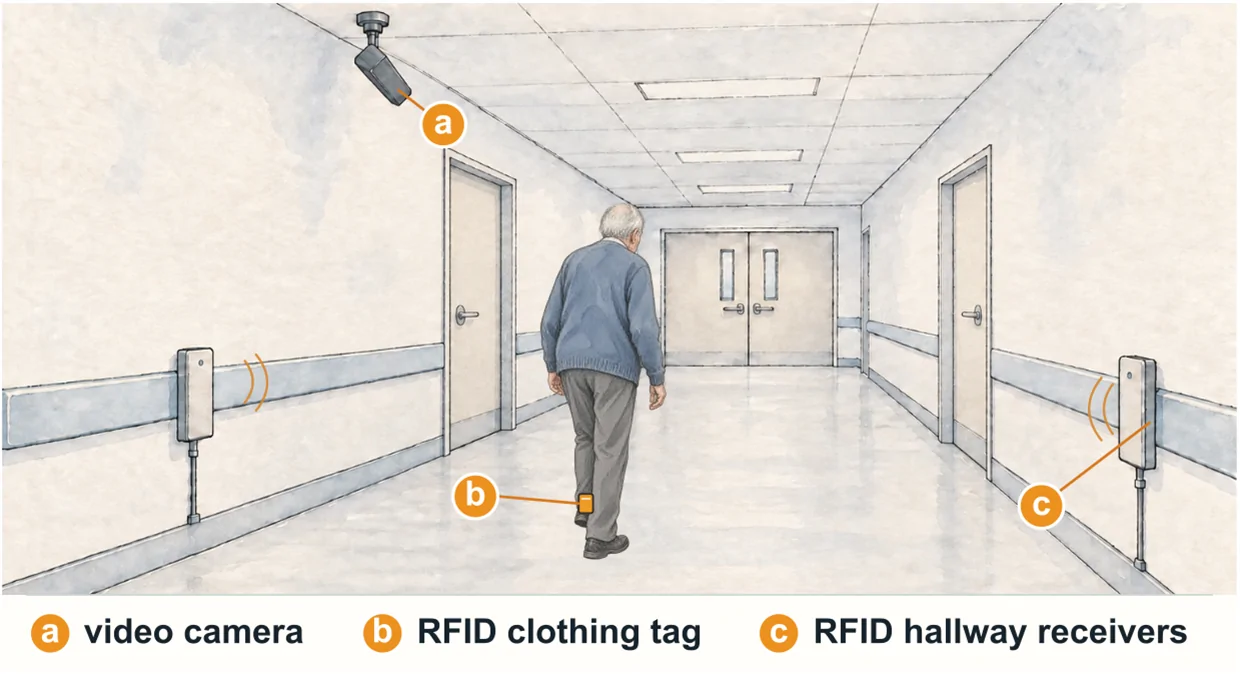
Hardware setup for unobtrusive hallway gait monitoring. The system used a ceiling-mounted RGB camera positioned above the walking path (a) and RFID-triggered recording (b: RFID clothing tag, c: RFID hallway receivers) to capture daily-life walking bouts in residential-care common areas. The schematic was created with assistance from OpenAI Codex (GPT-6.1 Sol), based on the monitoring setup described by Sabo et al. [9]. The authors reviewed the illustration for accuracy.

For each walking segment, the 3D pose sequence was converted to a fixed-length tabular representation by extracting 72 scalar gait features chosen to cover clinically interpretable domains of parkinsonian gait: spatiotemporal gait quantity and speed, variability, symmetry, joint range of motion, upper-limb motion, and postural stability (see S1 Appendix). Thus, “fixed-length” refers to the feature vector used by the model: walking bouts could differ in duration, but each bout was summarized by the same set of scalar statistics. These feature vectors were used as input to a TabPFN classifier [16], a transformer-based foundation model for tabular data. The model was trained to predict the UPDRS-III item 29 gait score on four publicly available datasets with UPDRS-Gait annotations (110 participants; 2953 walks) [17–21]. Because a score of 4 indicates inability to walk independently [22] and such individuals did not meet our inclusion criteria, we modeled the ordinal range 0–3.

The final model was retrained on all labeled walks and used to estimate gait severity for each walk in the unannotated T-SDU and T-LTC medication-analysis cohorts. For the medication analyses, the walk-level gait-severity score was the predicted UPDRS-Gait class.

### Statistical analysis

We assessed whether predicted gait severity scores were associated with within-participant changes after psychotropic medication dose reduction. For each medication class, we aggregated gait severity to daily means per participant (participant-day; one observation per participant per day). Using medication administration and dosing records, each participant-day was classified as either (1) baseline on-medication days (reference) or (2) within an acute dose reduction/cessation window, defined as 0–21 days after a ≥50% reduction in recorded daily dose relative to the most recent prior participant-day with a dose record, or after medication cessation. The 21-day window was chosen to reflect a short-term discontinuation period, consistent with placebo-washout discontinuation intervals reported for antipsychotics [23].

We fitted separate linear mixed-effects models per medication class in participants ever exposed to that class (excluding never-users), with fixed effects for time (weeks since each participant’s first observed day), dose-reduction/cessation state, centered age (years), and sex, and participant-specific random intercepts and random slopes for time to accommodate repeated measures. Models were fit using participant-days observed either while on medication or within the post-reduction window. All eligible participant-days from ever-exposed participants were retained to maximize precision and to accommodate unbalanced follow-up (i.e., differing numbers of observed days per participant).

As a complementary equal-weight participant-level summary, we additionally performed a paired analysis by computing, for each participant, the difference between mean daily gait severity during dose-reduction/cessation days and mean daily gait severity during on-medication days. This analysis gives each paired participant one contrast, regardless of the number of acute or on-medication participant-days contributed. Because this contrast requires observations in both states, the paired analysis was restricted to participants who contributed at least one on-medication day and at least one acute post-change day. Statistical significance was defined as α=0.05.

### Model validation and interpretability analyses

Because the medication-analysis outcome was model-derived, we evaluated model performance in T-SDU-PD, a labeled subset of T-SDU that was collected in the same hallway monitoring environment. Since T-SDU-PD is a sparsely sampled labeled subset of T-SDU selected and annotated by an expert annotator to cover the UPDRS-Gait severity range, it lacks the longitudinal medication-state coverage needed for medication-association and sensitivity analyses. Labeled external datasets remained available for model training, and T-SDU-PD participants were held out in five participant-stratified folds; all walks from a participant were assigned to a single fold.

To assess model interpretability, we computed model-agnostic SHAP values for the expected UPDRS-Gait score from the final TabPFN model [24]. SHAP values were computed for labeled T-SDU-PD examples using standardized TabPFN input features, with background samples from the full labeled training feature set defining the baseline prediction. High-contributing features were summarized using the feature names and categories listed in Table A in S1 Appendix.

### Medication-association sensitivity analyses

To evaluate robustness of the medication analyses, we refit pooled site-adjusted models using alternative post-reduction windows of 14, 28, 42, 56, and 84 days, and fitted lagged categorical models comparing on-medication days with 0–21, 22–56, and 57–84 day post-reduction bins. Lag bins with fewer than two exposed participants or five participant-days were flagged as insufficient and excluded from interpretation. We also quantified site imbalance and refit the primary 21-day model separately by site when sufficient exposed participant-days were available. Finally, to control for measured confounding factors, we refit the primary model with additional baseline clinical covariates (NPI and Katz, with an additional SIB-S sensitivity model when available).

## Results

### Dataset statistics and demographics

The dataset included 61 participants (Table 1), distributed across two centers: T-SDU (*n* = 49) and T-LTC (*n* = 12). Participants at T-SDU were younger on average (76.3 ± 7.7 years) than those at T-LTC (82.2 ± 6.4 years). A total of 2732 walks were recorded at T-SDU and 536 at T-LTC; participants at T-LTC were observed over a substantially longer period (median 195.5 [IQR 162.5–248.5] days) than those at T-SDU (median 32.0 [IQR 10.0–48.0] days). The proportion of males was higher at T-SDU (59.2%) than at T-LTC (25.0%). Participants at T-SDU had higher neuropsychiatric symptom severity (NPI 50.7 ± 22.0) than those at T-LTC (18.8 ± 19.2) and higher SIB-S scores (28.0 ± 14.4 vs. 22.2 ± 7.0), indicating more severe neuropsychiatric symptoms but less severe cognitive impairment on average. Katz scores showed minor variation between centers (2.1 ± 1.4 for T-SDU vs. 2.3 ± 1.7 for T-LTC).

**Table 1:** Participant characteristics stratified by site (*n* = 61).

Variable	T-SDU (n = 49)	T-LTC (n = 12)
Total walks, n	2732	536
Walks per participant, median [IQR]	41.0 [3.0–75.0]	28.0 [15.0–61.0]
Follow-up span, days, median [IQR]	32.0 [10.0–48.0]	195.5 [162.5–248.5]
Age, yrs	76.3 ± 7.7	82.2 ± 6.4
Male, %	59.2	25.0
NPI, points	50.7 ± 22.0	18.8 ± 19.2
Katz, points	2.1 ± 1.4	2.3 ± 1.7
SIB-S, points	28.0 ± 14.4	22.2 ± 7.0

Data presented as mean ± SD unless otherwise specified.

### Associations between psychotropic dose reduction and parkinsonian gait severity

Psychotropic exposure was common: 55 of the 61 participants (90%) were ever observed on an antipsychotic (AP) and 50 of 61 (82%) were ever observed on an antidepressant (AD) during follow-up. Analyses were performed separately for each medication class and restricted to ever-exposed participants (excluding never-users). Acute dose reduction/cessation events occurred in a subset of ever-exposed participants (AP: 15/55; AD: 20/50), contributing 451 and 550 walks, respectively, which were aggregated into 112 and 127 participant-day observations (Fig 2a). Among participants with an event, mean daily gait severity was 1.07 ± 0.78 on AP versus 0.82 ± 0.76 during dose reduction/cessation, and 1.12 ± 0.77 on AD versus 0.97 ± 0.74 during dose reduction/cessation (Fig 2b).

**Fig 2:**
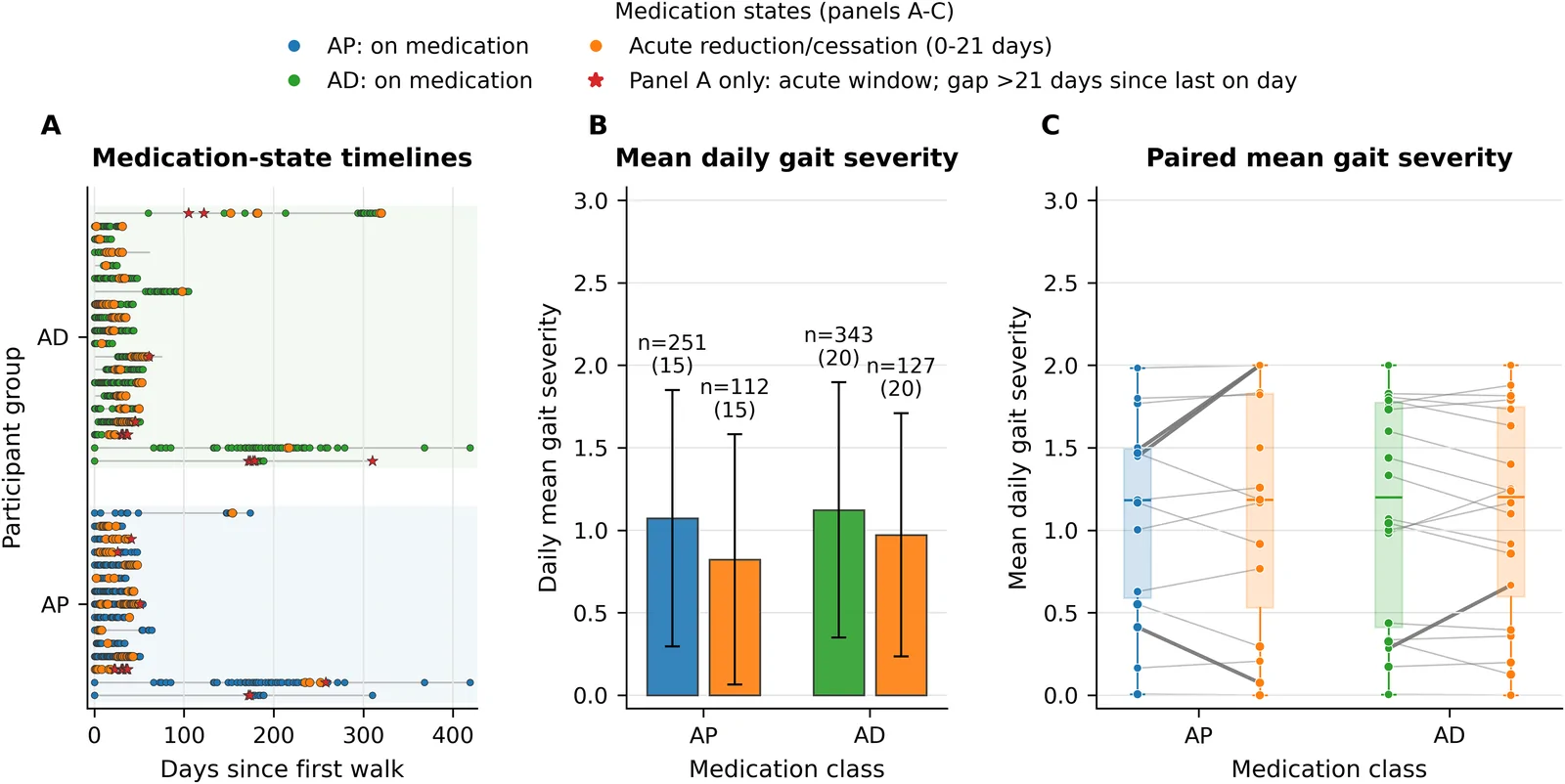
Gait severity across medication states for antipsychotic (AP) and antidepressant (AD) medications. Temporal visualization of dose reduction/cessation periods. Panel A: Longitudinal timeline of medication states for participants who experienced an acute dose reduction/cessation event. Blue circles represent days on AP, green circles represent days on AD, and orange circles represent days in the acute post-change window (0–21 days following a ≥50% dose reduction or cessation). Red stars indicate acute-window observations occurring >21 days after the most recent recorded on-medication observation (i.e., events inferred after gaps in observation). Panel B: Mean daily gait severity (± SD) on medication (blue/green) versus acute dose reduction/cessation days (orange) among participants with an acute event. Annotations give the number of participant-day observations, with the number of participants in parentheses. Panel C: Within-participant comparison of mean daily gait severity grouped by medication class. Dots and connecting lines represent individual participant means; overlaid boxplots display the median and interquartile range. Thick connecting lines highlight participants with an absolute change of |Δ|≥0.30 on the 0–3 predicted gait-severity scale, used as a descriptive threshold. Panel B averages across participant-day observations, giving greater weight to participants with more observed days in a given state, whereas Panel C displays paired participant-specific means, with each participant contributing one value per state.

To evaluate these differences while accounting for repeated measures, we fit separate linear mixed-effects models per medication class (Table 2). AP dose reduction/cessation was associated with lower gait severity compared with days on AP (β=−0.127, *p* = 0.022), whereas AD dose reduction/cessation was not (β=−0.060, *p* = 0.210). Because these models are fit on participant-day observations, participants with more observed days contribute more information; random effects model within-participant correlation but do not make each subject’s contribution equal. In contrast, a complementary equal-weight paired analysis among participants with both states (daily aggregated; one acute–on contrast per paired participant) found no significant mean within-participant change for either class (AP mean change (acute – on) = 0.031, approximate 95% CI [−0.114, 0.176], *p* = 0.653; AD mean change (acute – on) = −0.021, approximate 95% CI [−0.098, 0.056], *p* = 0.575). Fig 2c illustrates heterogeneous within-participant responses spanning both improvement and worsening (median change (acute – on): AP = 0.021; AD = −0.009), with a subset of participants crossing the descriptive threshold of |Δ|≥0.30 on the 0–3 predicted gait-severity scale.

**Table 2:** Associations of acute psychotropic dose reduction/cessation with gait severity.

	Antidepressant		Antipsychotic	
Predictor	β [95% CI]	*p*	β [95% CI]	*p*
Intercept	1.352 [1.129, 1.574]	<0.001*	1.358 [1.145, 1.570]	<0.001*
Time in study (weeks)	0.001 [−0.013, 0.015]	0.920	0.003 [−0.014, 0.020]	0.718
Acute dose reduction/cessation (0–21 days)^a^	−0.060 [−0.154, 0.034]	0.210	−0.127 [−0.235, −0.019]	0.022*
Sex (Male vs. Female)	−0.404 [−0.745, −0.063]	0.020*	−0.454 [−0.786, −0.122]	0.007*
Age (years, centered)	0.019 [−0.002, 0.039]	0.077	0.020 [0.000, 0.040]	0.054

**Note.** β = coefficient; CI = confidence interval; * *p* < 0.05.

^a^Daily-averaged (participant-day) observations from the acute post-change window (0–21 days) compared with days on medication; models were fit separately per medication class and restricted to ever-exposed participants.

### Model validation and interpretability

The labeled subset of T-SDU, T-SDU-PD, included 14 of 49 participants and 381 of 2732 walks. The TabPFN model achieved mean accuracy 0.562 ± 0.092, macro F1-score 0.487 ± 0.084, and quadratic-weighted κ=0.478±0.150 across five participant-stratified folds. These results indicate moderate validity for the ML-derived gait score in the dementia monitoring setting.

SHAP analysis of the final TabPFN model identified clinically interpretable contributors, including number of steps, walking speed, center-of-mass vertical displacement, arm swing amplitude, and elbow flexion (Fig 3; Table 3). These features span gait quantity and speed, postural motion, upper-limb motion, and lower-limb kinematics.

**Fig 3:**
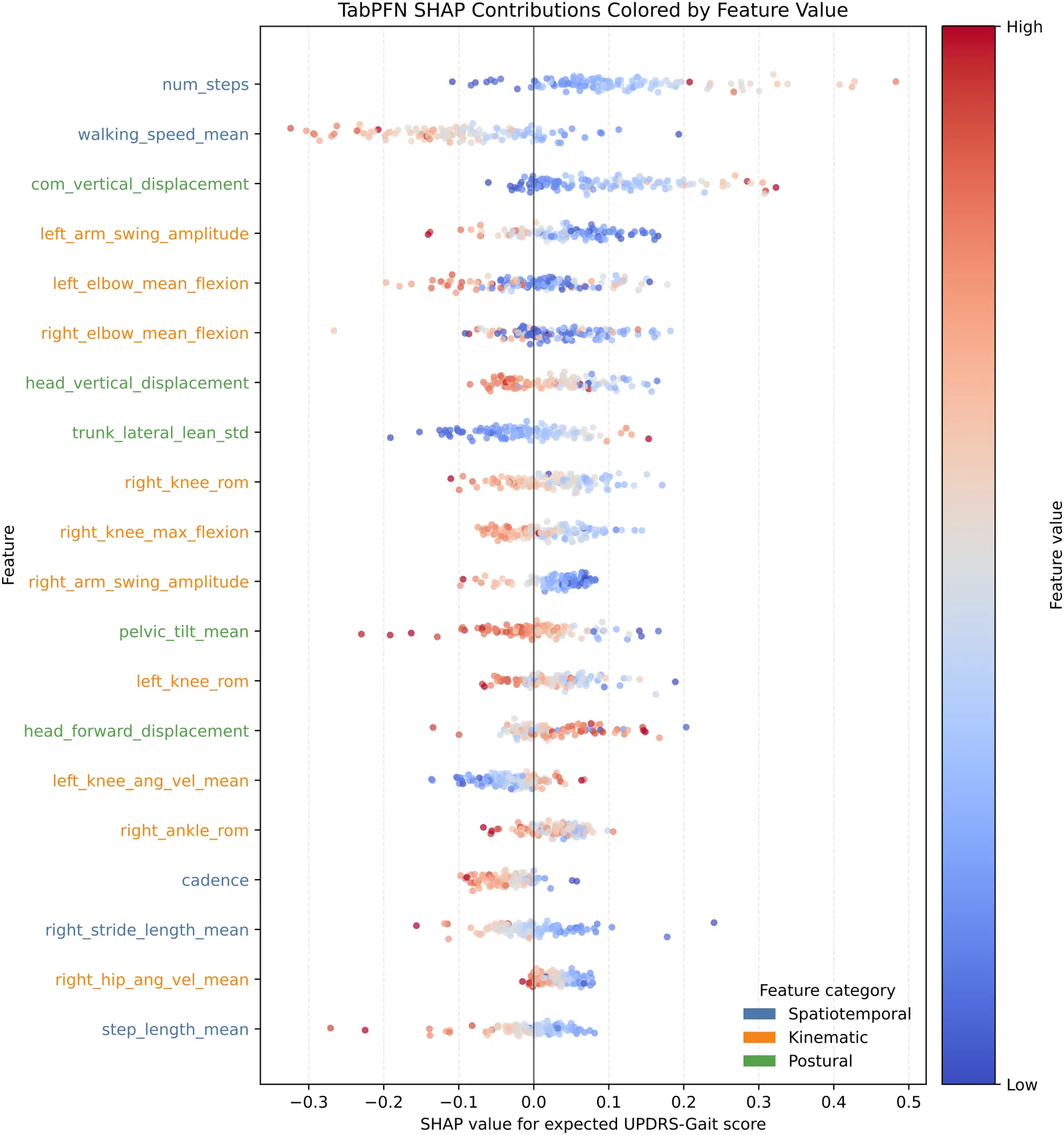
SHAP beeswarm plot for the final TabPFN model. Each point represents the SHAP contribution of one walk for a feature, with features ordered by mean absolute SHAP value and point colors indicating feature value (blue =low; red = high). Feature text colors indicate feature category (spatiotemporal, kinematic, postural, or symmetry). SHAP values were computed for the expected UPDRS-Gait score. The figure includes the twenty top contributing features, which did not include any symmetry-based features.

**Table 3:** Clinically interpretable features with high SHAP importance. Features are ordered by mean absolute SHAP contribution to the expected UPDRS-Gait score; full numeric values are provided in Table B in S1 Appendix.

Feature	Domain	Interpretation
Number of steps	Spatiotemporal	Given the relatively consistent hallway length, higher values may indicate shorter step length or shuffling gait
Walking speed	Spatiotemporal	Slower gait consistent with bradykinetic gait
Center-of-mass vertical displacement	Postural/gait dynamics	Reduced displacement may reflect hypokinetic gait, but it is inflated in our analyses due to a tilted SMPL/camera/world frame
Arm swing amplitude	Upper-limb motion	Reduced arm swing, a clinically recognizable parkinsonian hypokinetic gait feature
Elbow flexion	Upper-limb kinematics	May indicate altered reciprocal arm movement

Feature-family ablations showed that no single feature family fully drove performance. Dropping postural features produced the largest decline relative to the full model (macro F1-score change −0.043; κ change −0.036), whereas dropping spatiotemporal or symmetry features had smaller effects and dropping kinematic features slightly increased κ while reducing macro F1-score (Table C in S1 Appendix). Together, these analyses indicate that the model uses clinically plausible information from multiple gait domains and is not dominated by a single isolated feature family.

### Medication-association sensitivity analyses

Sensitivity analyses supported the direction of the main antipsychotic association while clarifying its limitations (S1 Appendix). In pooled site-adjusted models, AP dose reduction/cessation remained associated with lower gait severity across 14–84 day windows, becoming statistically significant for windows of 21 days and longer. AD estimates were negative and not statistically significant across the same binary windows. Lagged models suggested lower AP-associated gait severity in both the 0–21 and 22–56 day post-reduction bins; the 57–84 day bin had insufficient data. T-SDU had enough acute exposure for site-stratified models, whereas T-LTC contributed too few acute participant-days; the T-SDU-only estimates were similar in direction to the pooled estimates. Controlling for measured confounding factors by adding NPI and Katz, and additionally SIB-S where available, did not materially change the AP or AD conclusions.

## Discussion

This study evaluated whether unobtrusive vision-based gait monitoring can capture medication-linked changes in parkinsonian gait severity in residential dementia care. The system detected a modest participant-day–weighted association between acute antipsychotic dose reduction/cessation and lower predicted gait severity. Equal-weight participant-level paired analyses among participants observed in both states showed substantial heterogeneity and no consistent participant-average improvement. Antidepressant dose reduction/cessation showed no clear association, consistent with antidepressants being less frequently implicated in drug-induced parkinsonism than antipsychotics [1,25]. The complementary paired analysis is important for interpretation because it gives each paired event participant one contrast, whereas mixed-effects models weight information at the participant-day level. Improvements in some participants were offset by worsening in others. Thus, the results support feasibility and signal detection. They do not indicate a uniform population-level change.

We focused on antipsychotic and antidepressant dose reductions because both medication classes were common in the cohort and contributed enough post-change observations for within-participant analyses. The primary 0–21 day window was chosen to capture the best-observed acute period after a medication change: it provided 112 AP and 127 AD post-change participant-days while remaining close enough to the dose reduction/cessation event to limit dilution by later clinical changes. It is also consistent with short-term discontinuation or washout intervals used in antipsychotic studies [23]. Nevertheless, drug-induced parkinsonism is dose- and drug-dependent and can take weeks to months to resolve [26,27]. The sensitivity analyses therefore remain essential: AP estimates were directionally stable across wider 14–84 day windows, whereas AD estimates were not statistically significant in the binary-window analyses and showed a positive 22–56 day lagged estimate. These findings argue against overinterpreting the 21-day window as the only biologically relevant period.

Several limitations constrain causal interpretation. We did not capture the clinical rationale for dose reduction; common reasons may include suspected side effects, lack of efficacy, withdrawal attempts, behavioral change, or intercurrent illness. Consequently, gait could improve if dose reduction reverses drug-induced parkinsonism [28], worsen due to withdrawal phenomena [29], or change because medication adjustments coincide with behavioral or medical deterioration. The medication exposure definition grouped medications by class, limiting molecule-specific inference, and T-LTC contributed too few acute participant-days for stable site-stratified estimates. By excluding never-users and focusing on within-participant contrasts, we reduced confounding by baseline between-person differences. Residual time-varying confounding remains plausible.

Taken together, these findings suggest that continuous daily-life gait monitoring may be useful for identifying individuals with meaningful motor changes around psychotropic medication adjustments, especially in settings where routine clinical gait assessment is infrequent. The current results are unsuitable as a stand-alone basis for medication decisions. They motivate prospective studies with larger cohorts, medication-specific exposure definitions, documented reasons for medication changes, and longer follow-up to determine whether individual gait trajectories can help clinicians distinguish recovery from drug-induced parkinsonism, withdrawal-related worsening, and unrelated clinical change.

## Conclusion

In this two-center cohort study, we leveraged vision-based machine learning for longitudinal gait phenotyping around acute psychotropic dose reduction or cessation. Acute AP dose reduction/cessation was associated with lower gait severity in participant-day mixed-effects models, but equal-weight paired analyses did not show a consistent participant-average change, and participant-level responses were heterogeneous. The scalable vision-based pipeline could help flag individuals with meaningful within-participant changes for targeted clinical follow-up. These findings should be confirmed in a larger prospective study with longer follow-up and detailed documentation of the indication and timing of medication changes.

## Supporting information

S1 Appendix Supplementary methods and analyses. (PDF)
