# Supplementary material for: Assessment of change in drug-induced parkinsonism in daily-life gait using vision-based machine learning
Source: PLOS Digit Health. 2026 Oct 15;5(10):e0001780. doi: 10.1371/journal.pdig.0001780 (PMC13671658; doi:10.1371/journal.pdig.0001780)
Supplement: Supplementary file 1 [file pdig.0001780.s001.pdf]

# S1 Appendix. Supplementary methods and analyses

## Gait feature engineering

### Overview

A total of 72 scalar gait features were extracted from 3D pose trajectories for each walk across all datasets. These features quantify spatiotemporal gait characteristics, variability, symmetry, upper-limb motion, and postural stability. The features were standardized by removing the mean and scaling to unit variance.

### Feature list

We grouped features into the following conceptual categories:

- Spatiotemporal (e.g., step length, stride length, cadence, walking speed)
- Kinematic (e.g., hip, knee range of motion)
- Postural stability (e.g., total center-of-mass sway)
- Symmetry (e.g., step length symmetry index)

Table A summarizes all extracted features, grouped by category. For each feature we report a short description and measurement unit.

Table A: **Overview of extracted gait features.** Distance-based features were normalized by leg length.

| Category | Feature name | Description (unit) |
|---|---|---|
| Spatiotemporal | `left_stride_length_mean` | Mean left stride length |
| Spatiotemporal | `left_stride_length_std` | Standard deviation of left stride length |
| Spatiotemporal | `right_stride_length_mean` | Mean right stride length |
| Spatiotemporal | `right_stride_length_std` | Standard deviation of right stride length |
| Spatiotemporal | `step_length_mean` | Mean step length between consecutive feet |
| Spatiotemporal | `step_length_std` | Standard deviation of step length |
| Spatiotemporal | `step_width_mean` | Mean mediolateral distance between feet |
| Spatiotemporal | `step_width_std` | Standard deviation of step width |
| Spatiotemporal | `cadence` | Cadence (steps/min) |
| Spatiotemporal | `num_steps` | Total number of detected steps (count) |
| Spatiotemporal | `walking_speed_mean` | Mean forward walking speed |
| Spatiotemporal | `left_stride_time_mean` | Mean left stride time (s) |
| Spatiotemporal | `left_stance_percentage` | Mean left stance phase as percentage of gait cycle (%) |
| Spatiotemporal | `right_stride_time_mean` | Mean right stride time (s) |
| Spatiotemporal | `right_stance_percentage` | Mean right stance phase as percentage of gait cycle (%) |
| Spatiotemporal | `double_support_percentage` | Percentage of gait cycle in double support (%) |
| Kinematic | `left_hip_rom` | Left hip flexion/extension range of motion (deg) |
| Kinematic | `left_hip_mean_angle` | Mean left hip flexion angle (deg) |
| Kinematic | `left_hip_max_flexion` | Maximum left hip flexion angle (deg) |
| Kinematic | `right_hip_rom` | Right hip flexion/extension range of motion (deg) |
| Kinematic | `right_hip_mean_angle` | Mean right hip flexion angle (deg) |
| Kinematic | `right_hip_max_flexion` | Maximum right hip flexion angle (deg) |
| Kinematic | `left_knee_rom` | Left knee flexion/extension range of motion (deg) |
| Kinematic | `left_knee_max_flexion` | Maximum left knee flexion angle (deg) |
| Kinematic | `right_knee_rom` | Right knee flexion/extension range of motion (deg) |
| Kinematic | `right_knee_max_flexion` | Maximum right knee flexion angle (deg) |
| Kinematic | `left_ankle_rom` | Left ankle dorsiflexion/plantarflexion range of motion (deg) |
| Kinematic | `right_ankle_rom` | Right ankle dorsiflexion/plantarflexion range of motion (deg) |
| Kinematic | `left_arm_swing_amplitude` | Peak-to-peak forward displacement of left arm |
| Kinematic | `left_arm_swing_angular` | Angular swing amplitude of left arm (deg) |
| Kinematic | `left_arm_swing_frequency` | Arm swing frequency of left arm (Hz) |
| Kinematic | `right_arm_swing_amplitude` | Peak-to-peak forward displacement of right arm |
| Kinematic | `right_arm_swing_angular` | Angular swing amplitude of right arm (deg) |
| Kinematic | `right_arm_swing_frequency` | Arm swing frequency of right arm (Hz) |
| Kinematic | `left_elbow_mean_flexion` | Mean left elbow flexion angle (deg) |
| Kinematic | `left_elbow_rom` | Left elbow flexion range of motion (deg) |
| Kinematic | `right_elbow_mean_flexion` | Mean right elbow flexion angle (deg) |
| Kinematic | `right_elbow_rom` | Right elbow flexion range of motion (deg) |
| Kinematic | `left_hip_ang_vel_mean` | Mean absolute angular velocity of left hip (deg/s) |
| Kinematic | `right_hip_ang_vel_mean` | Mean absolute angular velocity of right hip (deg/s) |
| Kinematic | `left_knee_ang_vel_mean` | Mean absolute angular velocity of left knee (deg/s) |
| Kinematic | `right_knee_ang_vel_mean` | Mean absolute angular velocity of right knee (deg/s) |

| Category | Feature name | Description (unit) |
|---|---|---|
| Postural | trunk_lean_mean | Mean sagittal trunk lean angle (forward lean, deg) |
| Postural | trunk_lean_std | Variability of sagittal trunk lean (deg) |
| Postural | trunk_max_forward_lean | Maximum forward trunk lean angle (deg) |
| Postural | trunk_lateral_lean_mean | Mean absolute lateral trunk lean angle (deg) |
| Postural | trunk_lateral_lean_std | Variability of lateral trunk lean (deg) |
| Postural | head_forward_displacement | Mean forward displacement of head relative to pelvis |
| Postural | head_vertical_displacement | Mean vertical displacement of head relative to pelvis |
| Postural | pelvic_tilt_mean | Mean sagittal pelvic tilt angle (deg) |
| Postural | pelvic_tilt_std | Variability of sagittal pelvic tilt (deg) |
| Postural | pelvic_obliquity_mean | Mean absolute pelvic obliquity in frontal plane (deg) |
| Postural | pelvic_obliquity_std | Variability of pelvic obliquity (deg) |
| Postural | trunk_rotation_range | Range of horizontal trunk rotation (deg) |
| Postural | trunk_rotation_mean | Mean absolute horizontal trunk rotation (deg) |
| Postural | com_lateral_sway | Standard deviation of lateral center-of-mass position |
| Postural | com_vertical_displacement | Vertical excursion of center-of-mass |
| Postural | postural_stability_total_sway | Trunk sway magnitude from sagittal and frontal planes (deg) |
| Symmetry | stride_length_symmetry_index | Symmetry index of left–right stride length (%) |
| Symmetry | stride_length_asymmetry_ratio | Asymmetry ratio of left–right stride length (dimensionless) |
| Symmetry | step_length_symmetry_index | Symmetry index of left–right step length (%) |
| Symmetry | swing_time_symmetry_index | Symmetry index of left–right swing time (%) |
| Symmetry | stance_time_symmetry_index | Symmetry index of left–right stance time (%) |
| Symmetry | arm_swing_amplitude_index | Symmetry index of left–right arm swing amplitude (%) |
| Symmetry | arm_swing_amplitude_ratio | Asymmetry ratio of left–right arm swing amplitude (dimensionless) |
| Symmetry | hip_rom_symmetry_index | Symmetry index of left–right hip range of motion (%) |
| Symmetry | stride_time_cv | Coefficient of variation of stride time (left and right combined, %) |
| Symmetry | left_stride_time_cv | Coefficient of variation of left stride time (%) |
| Symmetry | right_stride_time_cv | Coefficient of variation of right stride time (%) |
| Symmetry | stride_length_cv | Coefficient of variation of stride length (%) |
| Symmetry | left_step_regularity | Step regularity of left leg (autocorrelation at step period) |
| Symmetry | right_step_regularity | Step regularity of right leg (autocorrelation at step period) |

## TabPFN model and T-SDU-PD validation

### Training procedure

Each walk is represented by a feature vector $\mathbf{x} \in \mathbb{R}^{72}$ and an ordinal UPDRS-Gait label $y \in \{0, 1, 2, 3\}$. The TabPFN classifier [1], a transformer-based foundation model for tabular classification, maps $\mathbf{x}$ to a distribution over classes $p(y \mid \mathbf{x})$, from which we obtain the predicted UPDRS-Gait score $\hat{y} = \arg\max_y p(y \mid \mathbf{x})$. We used the pretrained checkpoint `tabpfn-v2.5-classifier-v2.5_default-2` with default hyperparameters. Feature standardization parameters were estimated from training data within each validation fold and applied to held-out walks; the final model used standardization parameters estimated from all labeled training walks.

## Feature-family robustness

The main Results section reports SHAP feature importance and the top clinically interpretable contributors. Table B provides the corresponding numeric ranking for the top ten features. Feature-family ablations were performed by repeating the T-SDU-PD validation after dropping one conceptual feature family at a time. Performance remained in a similar range across ablations, although dropping postural features produced the largest decrease in macro F1-score and quadratic-weighted $\kappa$ (Table C).

Table B: **Top TabPFN features by mean absolute SHAP value.** Feature names match Table A.

| Rank | Feature | Category | Mean \|SHAP\| |
|---|---|---|---|
| 1 | num_steps | Spatiotemporal | 0.120 |
| 2 | walking_speed_mean | Spatiotemporal | 0.107 |
| 3 | com_vertical_displacement | Postural | 0.094 |
| 4 | left_arm_swing_amplitude | Kinematic | 0.058 |
| 5 | left_elbow_mean_flexion | Kinematic | 0.054 |
| 6 | right_elbow_mean_flexion | Kinematic | 0.052 |
| 7 | head_vertical_displacement | Postural | 0.050 |
| 8 | trunk_lateral_lean_std | Postural | 0.048 |
| 9 | right_knee_rom | Kinematic | 0.046 |
| 10 | right_knee_max_flexion | Kinematic | 0.041 |

Table C: **Leave-one-feature-family ablation results on the T-SDU-PD cohort.** Values are mean ± SD across five participant-stratified folds; Δ values are relative to the full model.

| Model variant | Accuracy | Macro F1-score | $\kappa_{\text{quadratic}}$ | $\Delta\kappa$ |
|---|---|---|---|---|
| Full model | 0.562 ± 0.092 | 0.487 ± 0.084 | 0.478 ± 0.150 | 0.000 |
| Drop spatiotemporal | 0.554 ± 0.051 | 0.479 ± 0.094 | 0.456 ± 0.159 | −0.022 |
| Drop kinematic | 0.566 ± 0.062 | 0.462 ± 0.053 | 0.499 ± 0.064 | 0.021 |
| Drop postural | 0.524 ± 0.065 | 0.444 ± 0.088 | 0.442 ± 0.229 | −0.036 |
| Drop symmetry | 0.556 ± 0.090 | 0.491 ± 0.096 | 0.487 ± 0.166 | 0.009 |

## Medication sensitivity and model-consistency analyses

Alternative post-reduction windows were evaluated in pooled site-adjusted mixed-effects models (Table D). For AP, all window estimates were negative, with statistical evidence for lower gait severity for the 21–84 day windows. For AD, all window estimates were negative but not statistically significant.

Table D: **Window sensitivity analyses for psychotropic dose reduction/cessation.** Estimates are pooled site-adjusted mixed-effects coefficients for acute dose reduction/cessation versus on-medication days.

| Medication | Window (days) | Participant-days | Event participant-days | $\beta$ | 95% CI | $p$ |
|---|---|---|---|---|---|---|
| AP | 14 | 858 | 92 | −0.097 | [−0.207, 0.012] | 0.080 |
| AP | 21 | 865 | 112 | −0.127 | [−0.235, −0.019] | 0.022 |
| AP | 28 | 865 | 118 | −0.146 | [−0.255, −0.036] | 0.009 |
| AP | 42 | 865 | 121 | −0.185 | [−0.296, −0.074] | 0.001 |
| AP | 56 | 865 | 126 | −0.209 | [−0.321, −0.097] | $< 0.001$ |
| AP | 84 | 865 | 127 | −0.188 | [−0.302, −0.074] | 0.001 |
| AD | 14 | 754 | 106 | −0.064 | [−0.158, 0.030] | 0.185 |
| AD | 21 | 761 | 127 | −0.060 | [−0.154, 0.034] | 0.210 |
| AD | 28 | 770 | 144 | −0.014 | [−0.112, 0.084] | 0.776 |
| AD | 42 | 779 | 158 | −0.015 | [−0.115, 0.085] | 0.768 |
| AD | 56 | 781 | 160 | −0.014 | [−0.114, 0.085] | 0.777 |
| AD | 84 | 781 | 160 | −0.014 | [−0.114, 0.085] | 0.777 |

In separate lagged categorical models, which compared on-medication days with 0–21, 22–56, and 57–84 day post-reduction bins, AP estimates were lower in the 0–21 day bin ($\beta = -0.184$, $p = 0.001$) and 22–56 day bin ($\beta = -0.561$, $p < 0.001$), whereas the 57–84 day bin had insufficient data (one participant-day). AD showed no evidence in the 0–21 day bin ($\beta = -0.025$, $p = 0.631$), a positive estimate in the 22–56 day bin ($\beta = 0.246$, $p = 0.027$), and insufficient data in the 57–84 day bin.

Site balance remained uneven: T-LTC contributed 536 rows, 196 participant-days, and 12 participants, whereas T-SDU contributed 2732 rows, 790 participant-days, and 49 participants. Site-stratified estimates could be fit for T-SDU (AP: $\beta = -0.139$, $p = 0.010$; AD: $\beta = -0.057$, $p = 0.248$), but T-LTC had too few acute participant-days for stable site-stratified inference.

Additional clinical adjustment did not materially change the primary pattern (AP: $\beta = -0.128$, $p = 0.021$ with NPI and Katz; $\beta = -0.132$, $p = 0.017$ with NPI, Katz, and SIB-S; AD: $\beta = -0.062$, $p = 0.196$ with NPI and Katz; $\beta = -0.069$, $p = 0.154$ with NPI, Katz, and SIB-S).

# References

[1] Hollmann N, Müller S, Purucker L, Krishnakumar A, Körfer M, Hoo SB, et al. Accurate predictions on small data with a tabular foundation model. Nature. 2025 Jan;637(8045):319-26.
